# Impact of an inducement to give high doses of amikacin and gentamicin on serum concentrations in critically ill patients with severe sepsis

**DOI:** 10.1186/2197-425X-3-S1-A402

**Published:** 2015-10-01

**Authors:** C Roger, B Louart, L Muller, JA Roberts, JY Lefrant

**Affiliations:** Nimes University Hospital, Nimes, France; University of Queensland, Brisbane, Australia

## Introduction

Low first-dose peak serum concentrations of amikacin and gentamicin are reported in intensive care unit (ICU) patients.^1^

## Objectives

The present study aimed at assessing the impact of giving high doses of amikacin (30 mg/kg) or gentamicin (8 mg/kg) in ICU patients with severe sepsis.

## Methods

Single-center observational study. All ICU patients with clinical indication of aminoglycosides were eligible.ICU physicians were encouraged to administer maximal recommended doses of amikacin and gentamicin (30 and 8 mg/kg, respectively). The first and subsequent doses and corresponding peak plasma concentrations were recorded. Guideline targets for serum concentrations were used with ≥60 and ≥30 mg/L for amikacin and gentamicin, respectively. A target pharmacokinetic/pharmacodynamic (PK/PD) ratio of 10-times the minimal inhibitory concentration (10xMIC) was also measured.

## Results

Sixty-three ICU patients (39 males, 68 ± 16 years, 75 ± 22 kg, 168 ± 8 cm, SAPS II = 43 ± 16) with severe sepsis and an indication for IV amikacin (n= 47) or gentamicin (n = 16), were included. Pulmonary, abdominal and urinary tract infections were diagnosed in 56 patients. Infection was confirmed in 37 (59%) patients. The target first-dose peak serum concentration was achieved in 37/63 patients (59%)(36/47 (77%) and 1/16 (6%) patient for amikacin and gentamicin, respectively). 59/63 (94%) patients achieved the PK/PD target using the MIC data that was available from 21 patients. However, the subsequent injection should be cancelled in nearly half of patients due to a too high trough, without renal function impairment.

## Conclusions

30 mg/kg amikacin and 8 mg/kg gentamicin doses led to adequate peak serum concentrations in 59% patients using guideline targets.

**Figure 1:**
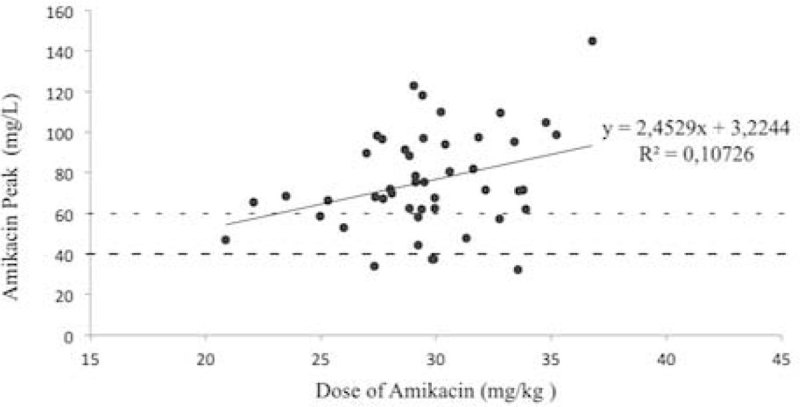
**Amikacin peak concentrations after the first dose.**
